# A Randomized Clinical Trial to Evaluate the Effects of Safinamide on Apathetic Non-demented Patients With Parkinson's Disease

**DOI:** 10.3389/fneur.2022.866502

**Published:** 2022-06-02

**Authors:** Jaime Kulisevsky, Saul Martínez-Horta, Antonia Campolongo, Berta Pascual-Sedano, Juan Marín-Lahoz, Helena Bejr-kasem, Ignacio Aracil-Bolaños, Andrea Horta-Barba, Arnau Puig-Davi, Javier Pagonabarraga

**Affiliations:** ^1^Movement Disorders Unit, Neurology Department, Sant Pau Hospital, Barcelona, Spain; ^2^Medicine Department, Universitat Autònoma de Barcelona (U.A.B.), Barcelona, Spain; ^3^Institut d'Investigacions Biomèdiques- Sant Pau (IIB-Sant Pau), Barcelona, Spain; ^4^Centro de Investigación en Red-Enfermedades Neurodegenerativas (CIBERNED), Cáceres, Spain; ^5^Neurology Department—Hospital Quirón Dexeus—Universitat Oberta de Catalunya (UOC), Barcelona, Spain

**Keywords:** Parkinson's disease, apathy, safinamide, RCT-randomized controlled trial, clinical trial

## Abstract

**Background:**

Apathy is highly prevalent and disabling in Parkinson's disease (PD). Pharmacological options for its management lack sufficient evidence.

**Objective:**

We studied the effects of safinamide on apathy in PD.

**Methods:**

Prospective, 24-week, two-site, randomized, double-blind, placebo-controlled, parallel-group exploratory study in non-demented PD on stable dopaminergic therapy randomized 1:1 to adjunct safinamide (50 mg/day for 2 weeks and 100 mg/day for 22 weeks) or placebo. The primary endpoint was the mean change from baseline to week 24 on the Apathy Scale (AS) total score. Secondary endpoints included changes in cognition, activities of daily living, motor scores, the impression of change, and safety and tolerability measures.

**Results:**

In total, 30 participants (active treatment = 15; placebo = 15; 80% showing clinically significant apathetic symptoms according to the AS) were enrolled, and included in the intention-to-treat analysis. Change in AS (ANOVA) showed a trend to significance [*p* = 0.059] mediated by a more marked decrease in AS score with safinamide (−7.5 ± 6.9) than with placebo (−2.8 ± 5.7). *Post-hoc* analysis (paired *t*-test) showed a significant positive change in the AS score between 12-week and 24-week [*p* = 0.001] only in the active group. No significant or trend changes were found for any of the secondary outcome variables. Adverse events were few and only mild in both treatment groups.

**Conclusions:**

Safinamide was safe and well-tolerated, but failed to provide evidence of improved apathy. The positive trend observed in the *post-hoc* analyses deserves to be studied in depth in larger studies.

**Trial Registration:**

EudraCT 2017-003254-17.

## Introduction

Apathy, is one of the more common and debilitating neuropsychiatric disturbances in Parkinson's disease (PD) (1, 2). It substantially contributes to reductions in quality of life, higher levels of care dependency, increased caregiver distress, and increased risk of developing dementia (2–5).

Apathy is manifested as a quantitative reduction of goal-directed activity in comparison to the person's previous level of functioning, which can be observed in behavioral, cognitive, emotional, or social dimensions (1, 6). Considering all the stages of PD, estimates of the prevalence of apathy range from 35 to 70%(1, 7).

While apathy highly overlaps along the course of the disease with symptoms of depression, anxiety, and cognitive impairment (8–11), it is a distinct neuropsychiatric syndrome (12) that can be properly identified using appropriate instruments (7, 13–16).

Apathy in PD is thought to mainly be due to the denervation of ascending dopaminergic pathways causing dysfunction of the prefrontal cortex-basal ganglia circuits (1, 6, 17) but other degenerated neurotransmitter systems can be compromised as well.

Among the behavioral complications of PD, apathy is likely the most underserved in terms of specific drug therapy. Very few high-quality randomized-control trials (RCTs) used apathy as an inclusion criterion (18). Two small-sized RCT in people with PD (PwP)–one with the dopamine agonist piribedil in PwP that turned apathetic after STN–DBS (19) and one with the anticholinesterase agent rivastigmine (20)—showed some positive results, the but evidence was not considered enough to qualify these compounds both as “efficacious” and useful' agents for the treatment of apathy in PD (18). Two studies using rotigotine for apathy in PwP were negative (21, 22), and one using 5-hydroxytryptophan observed positive effect on depression but not on apathy (23). Among non-RCT studies, rivastigmine failed to improve apathy in a 1-year open-label study in PD dementia (24), and positive effects in some non-motor symptoms, including apathy, were reported in open-label or *post-hoc* studies with rotigotine (25), pramipexole (17), istradefylline (26), and safinamide (27, 28).

Thus, there are no guidelines currently for managing apathy in PD (18, 29) and recommendations are limited to debatable expert opinion (1, 18, 29, 30). Hence, there is an urgent unmet need to adequately explore treatments to improve apathy in PD.

The dopaminergic system plays a core role in the regulation of goal-directed and motivating effortful behavior for reward, and its dysfunction has been proposed to play a crucial role in the etiology of apathy in PD (31). In this line, there is remarkable evidence regarding the involvement of the mesolimbic system and structures such as the nucleus accumbens—which play a central role in motivation—in the etiology of apathy in PD (1, 32, 33). However, dopaminergic replacement therapy generally has a partial or no effect on apathy in PD (19). Therefore, considering the role of other neurotransmitter systems involved in the normal functioning of the basal ganglia deserves to be taken into account in order to develop effective therapies.

Substantial evidence implicates the nucleus accumbens glutamine-to-glutamate ratio on the prediction of specific components of motivated behavior (34), and glutamine-to-glutamate ratio in the nucleus accumbens predicts effort-based motivated performance in humans (34). These arguments added to preliminary evidence from *post-hoc* and open-label studies showing some improvement in apathy in patients treated with safinamide (27, 28, 35) moved us to formally explore whether a therapeutic strategy using a drug targeting both, dopaminergic and glutamatergic systems, could help to ameliorate apathetic symptoms in PD.

Accordingly, in this study we explored the effects of Safinamide, a multimodal drug with a dual mechanism of action, dopaminergic (reversible mono amine oxidase-B inhibition) and non-dopaminergic [modulation of the abnormal glutamate release(cites)]. It has a predictable beneficial effect on motor fluctuations (35, 36) and was suggested to decrease non-motor symptom burden as well (27, 28). Safinamide has a good safety profile even in special group of PwP with psychiatric complications (37), and was not tested formally in a RCT for apathy in PD.

## Materials and Methods

### Study Design

This was a 24-week, randomized, double-blind, placebo-controlled, add-on, parallel-group study to assess the effect of safinamide on apathy in patients with PD conducted in two centers in Spain. Eligible PwP were randomized (1:1) to 24 weeks of oral treatment with either safinamide 50 mg/day (first 2 weeks) and 100 mg/day (22 weeks) or matching placebo, added to their current, stable PD medications that were to remain unchanged throughout the study.

### Sample and Assessments

#### Inclusion Criteria

Key inclusion criteria were: (1) non-demented PwP with a clinical diagnosis of PD according to the Movement Disorder Society (MDS) PD Criteria (38), aged 45–85 years; (2) Hoehn and Yahr Stage (39) of I to III (mild-to-moderate motor severity) at screening; (3) a total score ≥20 on the Montreal Cognitive Assessment scale (MoCA) (40); (3) scoring 1 or more on the Apathy Item of the Neuropsychiatric Inventory (NPI) (41); (4) clinical diagnosis of apathy as defined by Diagnostic Criteria for Apathy in Clinical Practice (42); (5) to be able to speak, read, and understand in the language in which the tests are written; (6) receiving treatment with dopaminergic therapy: levodopa (with or without entacapone) and/or dopamine agonists at a stable dose for at least 4 weeks prior to screening and for the duration of the study; (7) understand and sign the appropriate approved Informed Consent Form of the Study.

#### Exclusion Criteria

Key exclusion criteria were: (1) diagnosis of moderate-to-severe dementia associated with PD, according to the MDS criteria (43); (2) active psychosis or major hallucinations, severe depression or delirium; history of alcohol or drug abuse for 3 months prior to screening; (3) mental/physical/social condition that could preclude performing efficacy or safety assessments; (4) severe white matter disease, multiple lacunar infarcts, or signs of significant vascular changes on MRI; (5) clinically significant or unstable medical or surgical condition that would, in the opinion of the investigator, preclude participation to the study; (6) currently experiencing significant motor complications (moderate or severe wearing off defined as score >2 on Item 4.4 of MDS-UPDRS Part IV) or disabling dyskinesia (defined as score >2 on Item 4.2 of MDS-UPDRS Part IV) (44); (7) previous treatment with safinamide; (8) treatment with anticholinergic, antidopaminergic medication or acetylcholinesterase inhibitors; and (9) use of MAO-B inhibitors (e.g., selegiline, rasagiline) within 4 weeks prior to screening.

### Primary Efficacy Endpoint

The primary efficacy endpoint was the mean change from baseline to week 24 in the 14-item Starkstein Apathy Scale (AS) (45) total score (range 0–42; higher scores indicating more severe apathy).

### Secondary Endpoints

Key secondary endpoints were changed from baseline to week 24 in: (1) Parkinson's Disease–Cognitive Rating Scale (PD–CRS) total score; (2) Parkinson's disease–Cognitive Functional Rating Scale (PD–CFRS) total score; (3) NPI; (4) Hamilton Depression Rating Scale (HAM-D); (5) MDS–UPDRS motor subscale (Part III) total score; and (6) Parkinson's Disease Questionnaire (PDQ-39) total score; both (7) Patient's Clinical Global Impression of Change (P–CGI) of Apathy and (8) Clinical Global Impression of Change (CGI) of apathy, were administered at the final visit of the study. Ratings in the P–CGI and CGI were based on a Likert-type scale (0 = not assessed, 1 = very much improved, 2 = much improved, 3 = minimally improved, 4 = no change, 5 = minimally worse, 6 = much worse, 7 = very much worse); maximum score on the scales was 7. Safety and tolerability were assessed through adverse event (AE) reporting and physical examination, body weight and vital signs and electrocardiogram and laboratory test with hematology and biochemistry obtained at baseline, 4, 12, and 24 weeks' visits.

### Statistical Analyses

For the planned data analysis, a type 1 error of 5% for the primary hypothesis (alpha 0.05) was assumed. All the efficacy analysis were performed in the modified-intention to treat (ITT) population, therefore, all those subjects randomized and who received at least one evaluation visit were included. We also included all those subjects in the safety analysis who have been randomized and have taken at least one dose of study medication. As a method of imputation of missing values, the Last Observation Carried Forward (LOCF) method was used. The primary efficacy endpoint was the mean change in the AS total score from baseline to week 24. If there were no differences between groups in age, gender, and education, the statistical model to follow was a two-way ANOVA. If there were differences between groups in age, gender, or education, the statistical model to follow was an ANCOVA (if there are differences in a quantitative variable) or three-way ANOVA (if there are differences in a categorical variable). For the analysis of secondary variables, we applied the same model as that for the primary variable. Statistical analysis was performed using the statistical software package SPSS 19.0 for Windows (SPSS Inc., Chicago, IL).

### Sample-Size Calculation

Accepting an alpha risk of 0.05 and a beta risk of 0.2 in a two-sided hypothesis test, we calculated that a sample size of 18 subjects per group (*N* = 36) provided 80% power to detect a difference in mean change of the AS between safinamide and placebo. The SD was assumed in 9, and a dropout rate of 20% was expected among subjects who might discontinue study participation, require safinamide dose suspension or increase dopaminergic dosages.

### Ethics

This study (EudraCT 2017-003254-17) was approved by the local Ethics Committee which complies with the regulatory requirements and the Declaration of Helsinki. Written informed consent was obtained before any study procedures from all the patients. The data that support the findings of this study are available on request from the corresponding author. The data are not publicly available due to privacy or ethical restrictions.

## Results

Early termination of the study due to restrictions caused by the global COVID-19 pandemic precluded the recruitment of the planned sample size (*N* = 36). This was decided in accordance with the Ethics Committee and communicated to the Spanish regulatory authorities. While all the subjects who were active at the beginning of the restrictions were able to complete their pending visits in a timely manner, screening and recruitment of new subjects were stopped because of security reasons. Considerations favoring early termination instead of temporary suspension were: the exploratory nature of the study with the recruitment close to the planned sample size; the uncertainty in the duration of the mobility restrictions and accessing to Hospital facilities; and the relatively close caducity data of the supplied medication and placebo.

**Figure 1:**
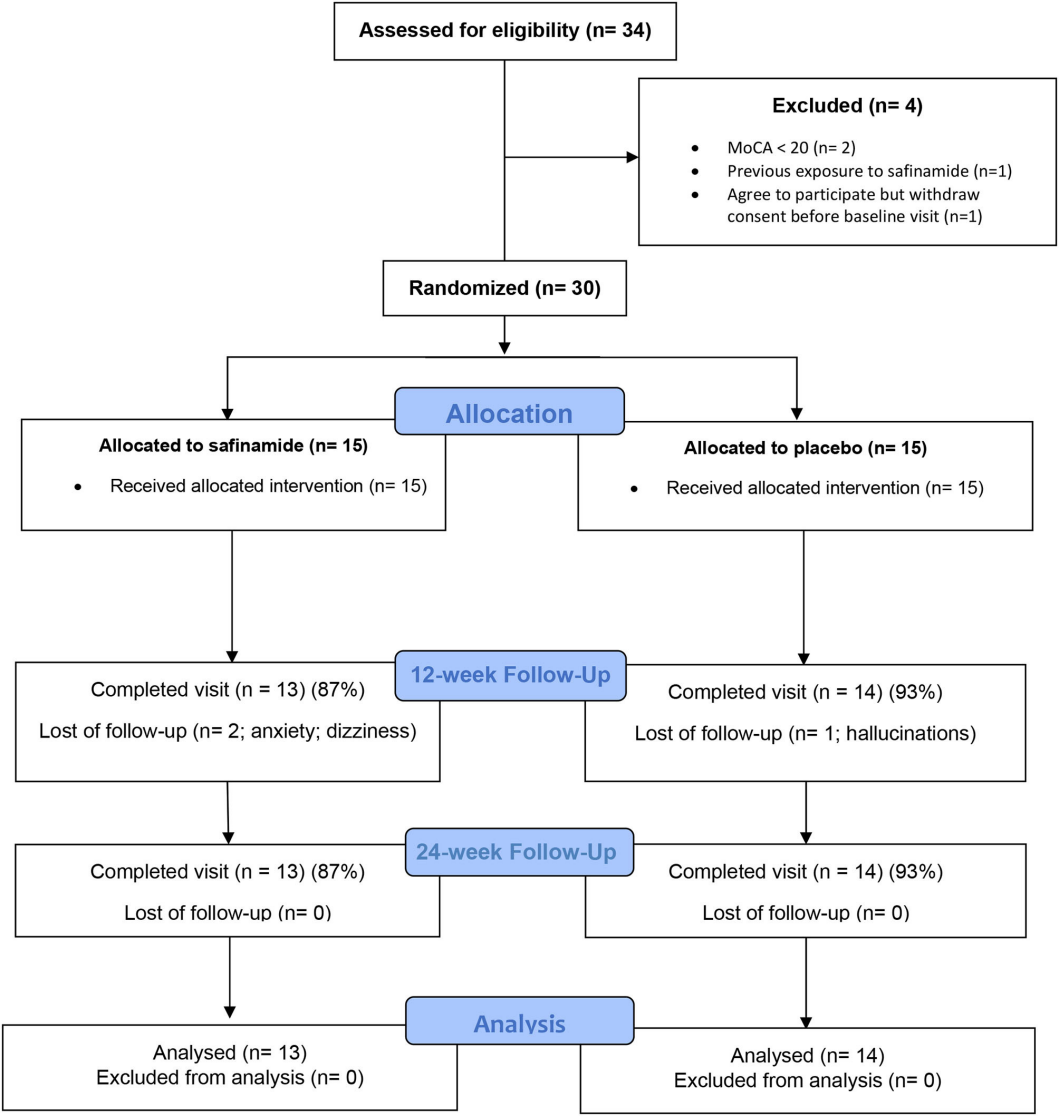
CONSORT diagram of participants in a study of safinamide for apathy in non-demented Parkinson's disease.

Screening, enrollment, and participation information is shown in Figure 1. Following screening (*N* = 34), eligible subjects (*N* = 30) were randomized to the safinamide or placebo groups. It supposed six fewer patients than the initially estimated as a total sample. The target dosage (100 mg/day) was achieved on all the participants in the safinamide group except in one subject who discontinued the study at visit 2 for mild dizziness. Other subject in the safinamide group complained of increase in anxiety and left the study at week 10. One subject on the placebo group left the study on week 16 due to hallucinations. According to the estimated dropout rate of 20% of the participants, the resulting sample was considered still valid in terms of sample size. Because of the planned ITT analysis in case of discontinuation, the results from all the 30 subjects participating in the study are reported.

The study sample consisted on 30 patients (mean age = 69.4 ± 9.9 years; mean disease duration = 53 ± 38.6; mean UPDRS-III = 29.9 ± 7.7; mean H&Y = 2 ± 0.4). As per inclusion criteria, all the patients scored ≥1 in the apathy sub-score of the NPI. After randomization, fifteen subjects were allocated to the active treatment (AT) arm and fifteen to the placebo arm. The main clinical and sociodemographic variables at baseline of the whole sample and of the two different treatment groups are described in Table 1.

**Table 1:** Sociodemographic and clinical characteristics of the entire sample and the two treatment groups at baseline.

	**Entire sample**		**Active treatment**	**Placebo**	
	**Mean ±SD**	**Range**	**Mean** **±SD**		***p***
Age	69.5 ± 9.8	44–84	66.7 ± 9.2	72.3 ± 10	0.149
Gender (f/m)	9/18	-	5/9	4/9	0.785
Education	12.3 ± 4.06	7–22	14.3 ± 4.6	10.2 ± 2.5	0.009
Disease duration	53 ± 38.6	8–134	57.8 ± 41.1	47.8 ± 36.6	0.512
UPDRS-III	29.9 ± 7.6	11–46	29.9 ± 8.3	29.9 ± 7.5	0.999
H&Y	2.1 ± 0.4	1–3	2.1 ± 0.3	2.1 ± 0.5	0.812
LEDD	609.2 ± 291.6	105–1,400	543 ± 213.8	631 ± 298.7	0.385
MoCA	25 ± 3	20–30	25.8 ± 3	23.9 ± 2.6	0.106
PD-CRS Total	89.1 ± 15.6	59–120	94.5 ± 16.2	81.1 ± 12.1	0.023
PD-CRS frontal-subcortical	59.44 ± 15.5	29–90	66.5 ± 14.6	51.7 ± 12.8	0.010
PD-CRS posterior-cortical	28.67 ± 6.2	22–30	28 ± 2.1	29.3 ± 8.7	0.571
PDQ-39	28.3 ± 17.1	2–59	25.4 ± 15.8	27.5 ± 17.1	0.742
NPI Apathy	4.1 ± 2.5	1–12	4.5 ± 2.9	3.8 ± 2	0.526
AS total score	19.5 ± 7.1	3–34	19.6 ± 7.2	19.3 ± 7.2	0.811
HAM-D	9 ± 5.1	1–23	9.6 ± 5.5	8.4 ± 5.1	0.594
Pharmacological treatment (%)					
*Antidepressants*	40.7	-	50	30.8	0.310
*Anxiolytics*	33.3	-	35.7	30.8	0.785
*Neuroleptics*	0	-	0	0	-
*Anticholinergics*	3.7	-	0	7.7	0.290
*IMAOs*	0	-	0	0	-
*Amantadine*	0	-	0	0	-
*Anticholinesterases*	7.4	-	7.1	7.7	0.957
*Methylphenidate*	0	-	0	0	-

*T*-tests showed absence of significant between-group differences in the main clinical variables associated with PD. Thus, no differences were found with respect to age, disease duration, H&Y stage, LEDD, pharmacological treatments, and UPDRS-III. Significant differences were found in education level [*t*_(30)_ = 2.81; *p* = 0.009], and in the PD–CRS total [*t*_(30)_ = 2.42; *p* = 0.023] and frontal-subcortical scores [*t*_(30)_ = 2.78; *p* = 0.010] with lower education level and PD–CRS scores in the placebo group. Despite baseline differences in the PD–CRS (used as secondary measure of the study), both the groups were equivalent in terms of global cognitive status measured at baseline with the MoCA. Accordingly, the proportion of patients scoring in the lower range of the PD–CRS was of 6.7% in the AT group and of 7.1% in the placebo group, and the proportion of patients scoring in the medium and higher range was of 93.3% in the AT group and of 92.9% in the placebo group, with no significant differences between the groups.

Both treatment groups showed at baseline an NPI apathy total score (frequency × severity) equal or higher than 1, and a mean AS above the clinical cut-off for apathy (AS ≥ 13), indicating that almost all the patients (75% in the AT groups; 85% in the placebo group; and 80% in the total sample) had clinically significant apathetic symptoms according to the AS, with no differences between groups in the proportion of this prevalence (×2 = 0.361).

### Primary Efficacy Endpoint Analysis

**Figure 2:**
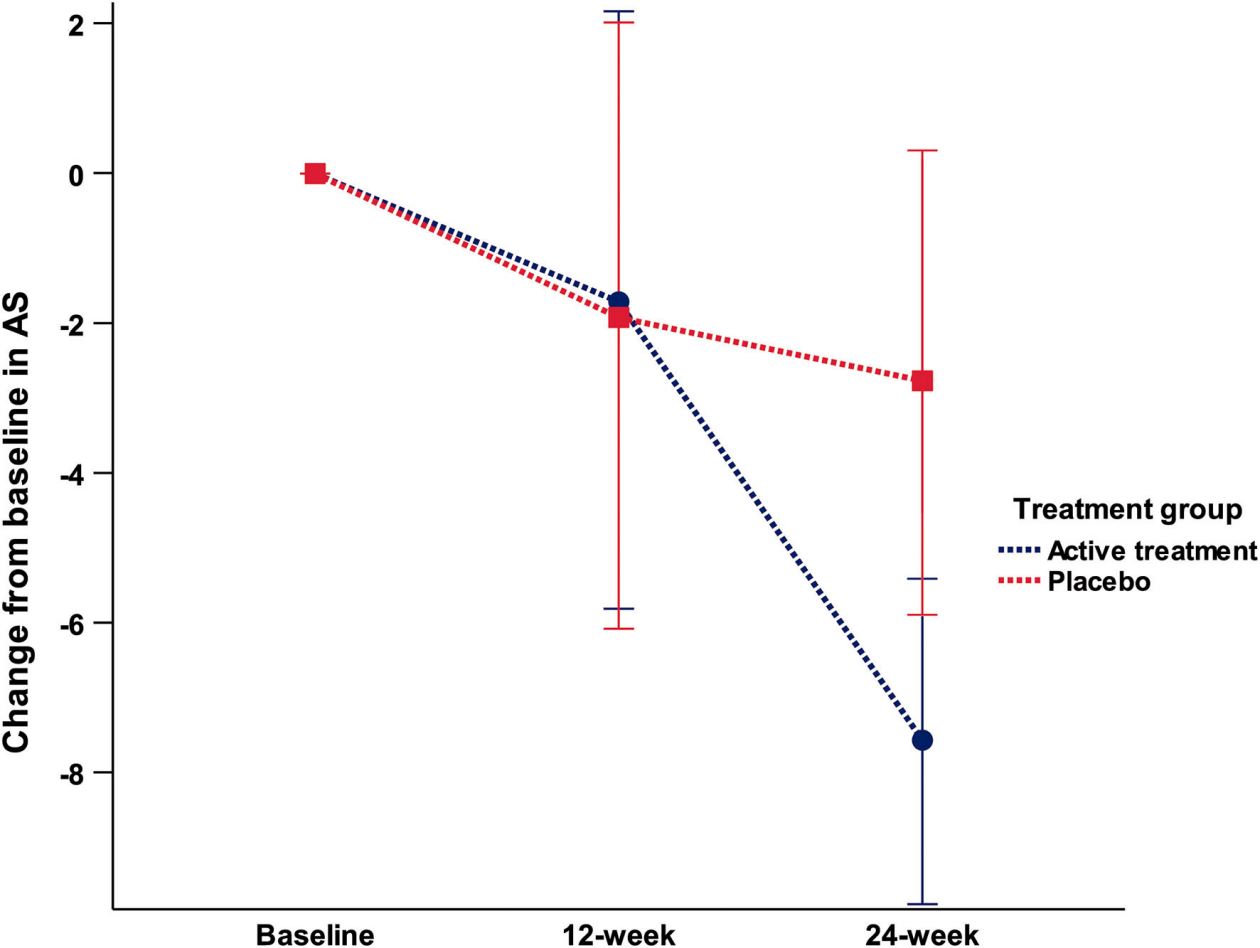
Mean change from baseline in the Apathy Scale in each consecutive visit.

Repeated measures ANOVA applied to explore the primary outcome measure (change in the AS score between 24-week and baseline) showed a trend to a significant group × time interaction [*F*_(1, 29)_ = 3.06; *p* = 0.059]. *Post-hoc* analysis showed that this effect was mediated by a more marked, and nearly significant decrease on the AS score in the AT group [*t*_(30)_ = −1.95; *p* = 0.062]. Thus, the mean change from baseline at 24 week was of −7.5 ± 6.9 in the AT group and of −2.8 ± 5.7 in the placebo group. As depicted in Figure 2, this effect was observed at 24 week in the AT group, while equivalent scores were obtained in the two groups at baseline and at 12 week.

Paired *t*-test within each group showed that in the AT group, no differences existed between baseline and 12-week AS score [*t*_(13)_ = 1.03; *p* = 0.318], but a significant difference was found between 24 week and 12 week [*t*_(13)_ = 4.22; *p* = 0.001], and between 24 week and baseline [*t*_(13)_ = 4.06; *p* = 0.001]. In the placebo group, no significant differences were found between visits.

**Figure 3:**
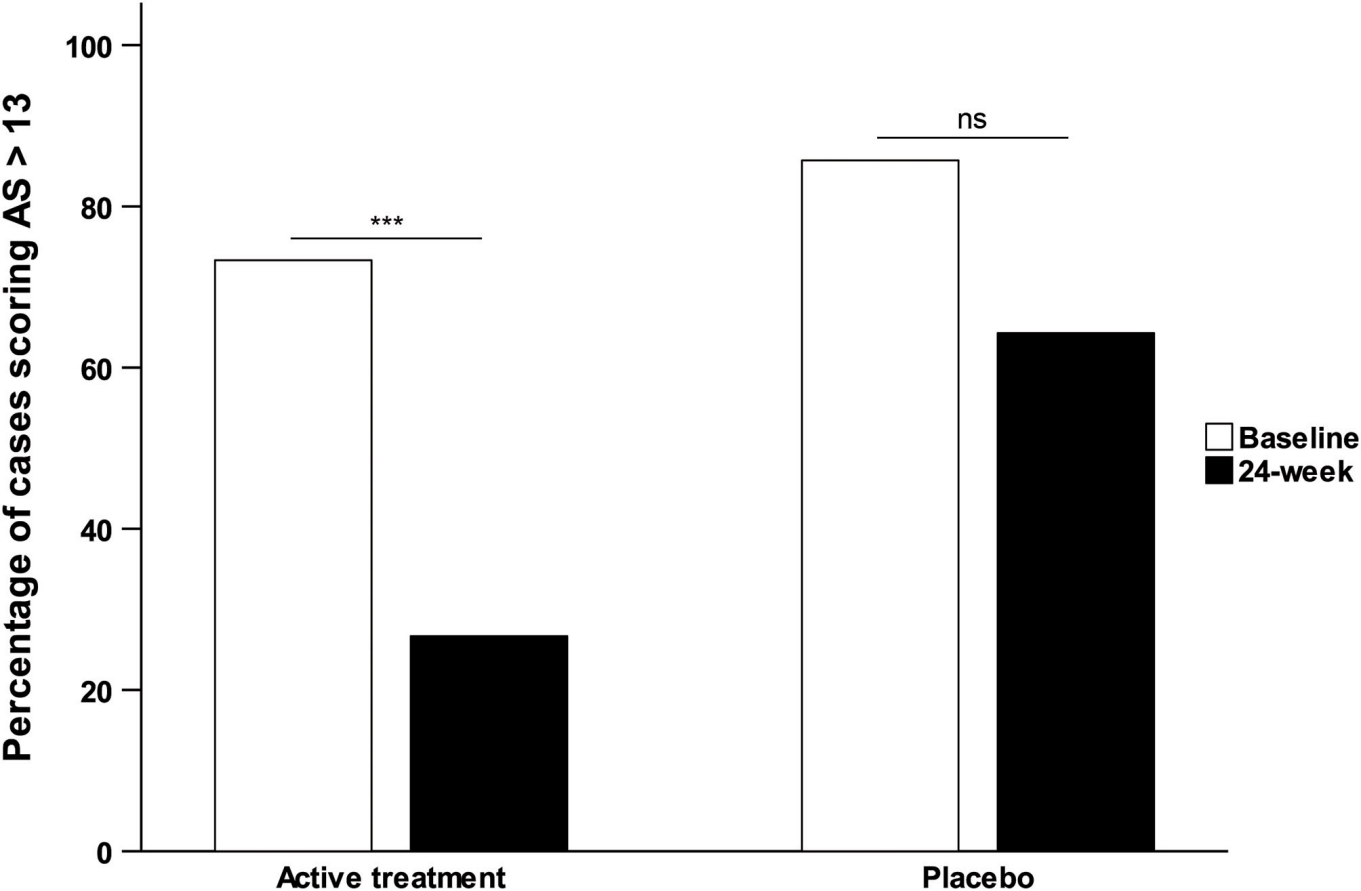
Percentage of participants scoring in the clinical range for apathy based on the Apathy Scale.

When analyzing the change from clinically relevant apathetic symptoms at baseline (AS > 13) to non-apathy (AS < 14) at 24 week, we observed that the significant decrease in the mean apathy severity score occurred in 46.6% of the subjects in the AT group compared with just 21.4% of those in the placebo group. This difference in the rate of conversion from clinically relevant apathy to non-apathy was significantly different between groups (*x*^2^ = 0.042; Figure 3).

### Secondary Efficacy Endpoints Analysis

#### Neuropsychiatric Inventory (NPI)

**Figure 4:**
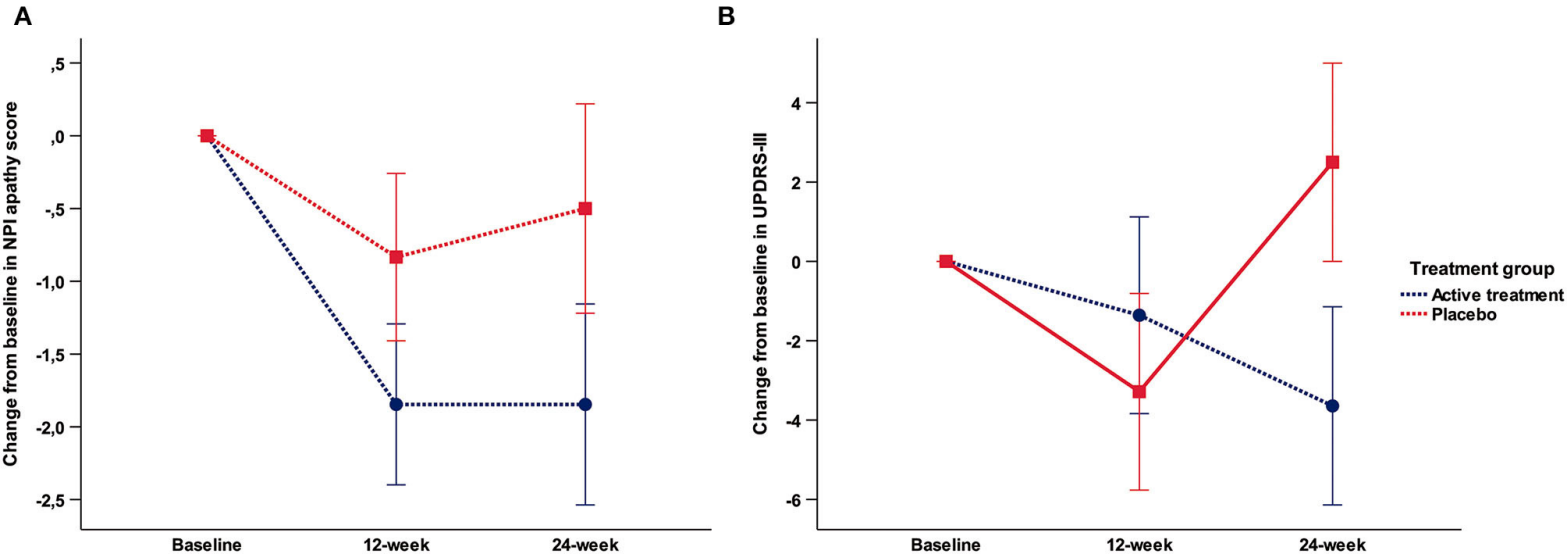
**(A)** Mean change from baseline in the NPI total apathy score; **(B)** Mean change from baseline in the UPDRS-III.

Repeated measures ANOVA showed no significant effects between groups and visits in the NPI total score for apathy (frequency × severity). However, as depicted in Figure 4A, *post-hoc t*-test comparison showed a trend to significance [*t*_(30)_ = −2.06; *p* = 0.053] at 24-week mediated by a mean change from baseline of −1.9 ± 2.2 points in the AT group compared with 0 ± 2.7 in the placebo group. No effects were found with respect to the other neuropsychiatric symptoms covered with the NPI. The statistics for the primary and secondary efficacy endpoints analysis are described in Table 2.

**Table 2:** Primary and secondary outcome measures analysis.

	**Active treatment**	**Placebo**	**Difference AT - Placebo**	
	**Mean ±SD**	**Mean ±SD**	**Estimate (95% CI)**	***p***
AS total score	−7.5 ± 6.9	−2.8 ± 5.7	−4.71 (−9.68 to 0.25)	0.062
NPI apathy	−1.9 ± 2.2	0 ± 2.7	−1.92 (−3.88 to 0.02)	0.053
PD-CRS total score	1.5 ± 8.9	−4.6 ± 10.6	6.21 (−1.42 to 13.8)	0.306
PD-CFRS	−0.5 ± 2.5	−0.1 ± 2.1	−0.33 (−2.24 to 1.57)	0.722
HAM-D	−1.5 ± 6.6	−0.9 ± 4.1	−0.57 (−4.85 to 3.7)	0.786
UPDRS-III	−3.6 ± 8	2.5 ± 10.5	−6.14 (−13.4 to 1.11)	0.094
PDQ-39	−5.6 ± 19.1	−0.6 ± 12.5	6.95 (−18.6 to 6.1)	0.312
P-CGI	4.08 ± 2.1	4.5 ± 1.9	−0.46 (−2.98 to 1.15)	0.562
P-CGI-QOL	3.08 ± 2.1	2.52 ± 2.1	0.66 (−1.03 to 2.35)	0.428
CGI	4 ± 1.5	3.5 ± 1.5	0.42 (−1.34 to 2.19)	0.607

#### UPDRS-III

No significant effects neither trend were found in the repeated measures ANOVA. *Post-hoc t*-test comparisons showed a trend to significance [*t*_(30)_ = −1.73; *p* = 0.094] at 24 week mediated by a mean change from baseline of −3.64 ± 8 in the AT group compared with 2.5 ± 10.5 in the placebo group (Figure 4B).

#### Other Secondary Endpoints

No significant effects or trends were found in the repeated measures ANOVA and *post-hoc* comparisons focusing on cognitive performances (PD–CRS), cognitive-functional status (PD–CFRS), quality of life (PDQ-39), and patient and clinical impression of change (P–CGI, P–CGI–QOL, and CGI).

### Safety and Tolerability

Safinamide at the doses of 50 and 100 mg/daily was safe and well-tolerated, and no major or unexpected safety concerns were identified. As reported, early discontinuation occurred in the three patients. One on the safinamide left the study due to mild dizziness at visit 2 without being scaled to receive the 100 mg/day dose, and the other due to increase in anxiety who left the study on week 10 while on 100 mg/day (Table 3). The one belonging to the placebo group left the study on week 16 due to hallucinations. No differences were found in vital signs and electrocardiogram, body weight and laboratory test, neither between groups in the baseline visit, nor in the successive follow-ups.

**Table 3:** Adverse events.

	**Visit (Week)**	***N* cases/%**	**Dosage**	**Study group**
Mild dizziness	4	1/3.33%	50 mg	Active
Anxiety	10	1/3.33%	100 mg	Active
Visual hallucinations	16	1/3.33%	100 mg	Placebo

## Discussion

This is the first RCT study exploring the effects of safinamide in non-demented patients with PwP, and one of the few prospective PD studies in which apathy was an inclusion criterion and the primary outcome.

The results of the study are positive in terms of safety, but negative in terms of the effect of Safinamide on apathy. Nevertheless, the results show a tendency toward static significance that we believe deserves consideration. Thus, the addition of safinamide in subjects with PD with significant apathy is well-tolerated and may result in a discrete beneficial effect observed in this study in the form of a trend toward significance. Although only reaching a trend to significance in the primary analysis, a beneficial effect of safinamide in comparison to placebo was observed between weeks 12 to 24 in the AT group in the *post-hoc* analysis. This was accompanied by a significant change favoring safinamide in the proportion of subjects moving from clinically significant apathetic symptoms at baseline to not clinically relevant symptoms at the end of the study. No relevant changes were found for any other explored variable, although in consistence with the objective of the study, the only additional statistical trend was a reduction from baseline in the mean NPI apathy score.

In addition to not having observed a statistically significant effect in the primary analysis, a number of lessons supporting further research of safinamide in PD-related apathy can be collected from this exploratory study. The temporal curve showed a trend to significance between weeks 12 and 24 in the safinamide group observed in the exploratory *post-hoc* analysis suggests that the beginning of the eventual positive effect of safinamide can be a delayed one. It is possible that a more consistent effect could have been observed with a longer follow-up and a larger number of patients. Importantly, these positive signals were detected only for the main variable and were not related to motor, mood, or cognitive changes. At last, safinamide was well-tolerated in a cohort of subjects with PD not selected for having levodopa-related fluctuations.

Besides not reaching the planned sample size, other factors related both with the pathogenesis of the apathy syndrome in PD and the characteristics of the tested drug, could have contributed to the modest benefit of associated with safinamide in our study.

Although apathy is highly prevalent in PD from its early stages, the exact pathogenesis of apathy in PD are partially understood at present (46), being likely a combination of progressive alteration of dopaminergic pathways (43, 47), brain atrophy in strategic reward nodes (24) with impaired incentive processing (33), synergistically acting alpha-synuclein and Alzheimer's disease (AD) protein aggregates and increased burden of vascular and inflammatory changes (48) that may limit the response to the pharmacological treatment (1, 49).

While partial correction of an altered neurotransmission may not suffice for apathy to significantly improve in PD, safinamide may have exerted a positive effect on dopamine-dependent apathetic symptoms. Still, considering that its action is not stronger than that of other dopaminergic agents that showed uneven results in improving apathy (49), other factors might concur to explain the partial response of apathy seen in this study.

A glutamate hypothesis for apathy arises from drug trials that suggests a link between the glutamatergic system and apathy symptoms in psychiatric and neurodegenerative diseases other than PD (50–52). While memantine, an agent that blocks the effects of pathologically elevated levels of glutamate, seems not to influence apathetic symptoms in AD, mibampator, a glutamate receptor potentiator, significantly improved apathy in a RCT in AD (50). On this basis, the dual action of a drug that reinforces dopaminergic transmission and blocks the effects of pathologically elevated levels of glutamate, may conceivably improve the synaptic connectivity and trigger the functional recovery of damaged neuronal network, which is typical of apathy.

In this line, blockade of sodium channels and modulation of calcium channels that is the base of the antiglutamatergic activity of safinamide, is not expected to be complete below dosing of 100 mg/day, which were not achieved until the third week of the study. This could explain the significant but delayed reduction in the mean apathy scores compared with the placebo observed in the *post-hoc* analysis of the second half of our study. Future studies should explore whether higher doses of safinamide and/or more prolonged treatment period have a significant clinical effect.

Consistent with the good safety profile of the drug observed in phase III and large-sample observational studies (37), almost all the apathetic subjects randomized to safinamide treatment completed the study, with a dropout rate of just 13% (two participants). Safinamide was generally well-tolerated over 24 weeks by patients who were receiving polypharmacy without substantial differences in the number or severity of adverse events compared with the placebo. Particularly, adding safinamide in apathetic patients did not worsen motor status, cognition and other important behavioral aspects including mood, hallucinations, or impulse control behavior.

A major strength of our study is that we selected patients accomplishing clinical criteria for apathy and tried to generate high-quality data using a validated instrument as primary outcome to address an important unmet in PD. Consequently, the average apathy rating scale scores obtained at baseline in the AS reflects a PD population with clinically significant apathy. Nevertheless, being apathy scores above the cut-off of apathy (45), they were not in the high range. This may be partially explained by the exclusion of demented patients and the diminished motivation of severely apathetic patients for participating in a research study.

Main limitation of our study was its early termination that precluded the recruitment of the planned sample, and possibly, reaching statistical significance in the primary objective. Nevertheless, our results provide valuable information to inform the design of future trials. A case for a possible favorable response, with a delayed initiation of action and a conceivable more consistent benefit in improving apathy with longer duration of treatment, can be made based on our data.

## Data Availability Statement

The raw data supporting the conclusions of this article will be made available by the authors, without undue reservation.

## Ethics Statement

The studies involving human participants were reviewed and approved by ERB Hospital de la Santa Creu i Sant Pau. The patients/participants provided their written informed consent to participate in this study.

## Author Contributions

JK and SM-H: conception and design of the study. JK, SM-H, BP-S, AC, JM-L, HB-k, IA-B, AH-B, and JP: recruitment of participants and assessments. SM-H and AP-D: data analysis. JK, SM-H, and JP: interpretation, draft manuscript, and review. All authors contributed to the article and approved the submitted version.

## Funding

This work was partially supported by CERCA and CIBERNED funding, and grants from la Marató de TV3 (grants #2014/U/477 and #20142910), Fondo de Investigaciones Sanitarias (FIS) from the Instituto de Salud Carlos III (ISCIII), and Fondo Europeo de Desarrollo Regional (FEDER) grants #PI15/00962 and #PI18/01717. Drug and placebo treatments were facilitated by the Zambon Italy that also provided an unrestricted grant for an independent Investigator-Initiated study. The funders were not involved in the study design, collection, analysis, interpretation of data, the writing of this article or the decision to submit it for publication.

## Conflict of Interest

JK has received honoraria for advisory boards or lecturing from: Teva, Zambon, UCB, Bial, General Electric, Sanofi, and Roche. JP has served on advisory or speakers' boards, and received honoraria from: UCB, Zambon, AbbVie, Italfarmaco, Allergan, Ipsen, Bial. The remaining authors declare that the research was conducted in the absence of any commercial or financial relationships that could be construed as a potential conflict of interest.

## Publisher's Note

All claims expressed in this article are solely those of the authors and do not necessarily represent those of their affiliated organizations, or those of the publisher, the editors and the reviewers. Any product that may be evaluated in this article, or claim that may be made by its manufacturer, is not guaranteed or endorsed by the publisher.
